# Multistructure index characterization of heart rate and systolic blood pressure reveals precursory signs of syncope

**DOI:** 10.1038/s41598-017-00354-x

**Published:** 2017-03-24

**Authors:** Danuta Makowiec, Beata Graff, Zbigniew R. Struzik

**Affiliations:** 1 Institute of Theoretical Physics and Astrophysics, The University of Gdańsk, Wita Stwosza 57, 80-308 Gdańsk, Poland; 2 Department of Hypertension and Diabetology, Medical University of Gdańsk, M. Sklodowskiej-Curie 3a, 80-210 Gdańsk, Poland; 3 RIKEN Brain Science Institute, 2-1 Hirosawa, Wako-shi, 351-0198 Japan; 4 Graduate School of Education, The University of Tokyo, 7-3-1 Hongo, Bunkyo-ku, Tokyo 113-0033 Japan

## Abstract

Recurrent syncope — abrupt loss of consciousness — can have a serious impact on patients’ quality of life, comparable with chronic illnesses. Vasovagal syncope (VVS) is a specific reflex syncope, in which an inappropriate reaction of the autonomic nervous system (ANS) plays a key role in the pathophysiology. In syncope diagnosis, an ideal diagnostic method should positively identify vasovagal sensitive patients, without the need to perform a specialised head-up tilt table (HUTT) test. We apply a novel methodology of multistructure index (MI) statistics for seamlessly evaluating the size spectrum of the asymmetry properties of magnitudes of neural reflexes responsible for maintaining the homeostatic dynamics of autonomic control. Simultaneous evaluation using the MI of the effects on heart rate and blood pressure involved in achieving homeostasis of contrasting properties of the dynamics of slow and fast neural regulation reveals a clear distinction between vasovagal patients and healthy subjects, who are/are not susceptible to spontaneous fainting. Remarkably, a healthy cardiovascular response to the HUTT test is indeed evident prior to the test, making the MI a robust novel indicator, clearly distinguishing the cardiovascular autonomic regulation of healthy people from that of vasovagal patients without the need to perform an actual HUTT test.

## Introduction

The physiological mechanisms responsible for heart rate accelerations and decelerations and for rises and falls in blood pressure are distinct^1^. The autonomic nervous system (ANS) mediates these processes to maintain the body’s homeostatic state^2^. Methods evaluating changes in the time intervals between consecutive heart contractions, called heart rate variability (HRV)^3^, and beat-to-beat variation of the level of blood pressure, called blood pressure variability (BPV)^4^, have been proposed to assess the ANS control. These approaches have been successful both in extending our knowledge about cardiovascular regulatory mechanisms and also in enabling the design of clinically important measures of risk prediction^3^.

Disorder of the autonomic regulation is considered to be the main reason for vasovagal syncope (VVS) — a transient loss of consciousness resulting from the orthostatic stress^5–8^. The upright posture induces downward displacement of about 500 to 1000 ml of blood and the major part of this redistribution occurs within the first ten seconds. During the following minutes, an additional 700 ml of fluid gradually relocates into the interstitial space. Therefore, various regulatory mechanisms have to be involved in maintaining a proper blood supply for the vital organs, including the brain. A decrease in blood pressure is sensed by arterial baroreceptors, which first results in the decreased activation of the vagal branch of the ANS and in the subsequent rise of the heart rate within one or two following heartbeats. Then, after 1–3 seconds the rise in the sympathetic ANS branch activity increases both the heart rate and heart muscle contractility, and induces the constriction of small arteries and arterioles^1, 6^. In addition, local vasoconstrictor mechanisms are activated. In contrast, vasovagal patients in the upright position may experience vasodilation and/or bradycardia resulting in hypotension, a decrease in brain perfusion, and finally, the loss of consciousness^7^.

Although vasovagal syncope is very common, its pathophysiology is not fully understood. Several concepts have been developed, including ventricular theory, baroreflex dysfunction theory, reduced blood volume theory, neurohumoral theories and active vasodilation theory. The roles of respiration and cerebral blood flow deregulation have also been considered. However, many of the attempts made to explain the various mechanisms involved in vasovagal syncope have led to contradictory results^5^.

A simple and non-invasive method, referred to as a head-up tilt test (HUTT), is usually used to provoke vasovagal syncope in laboratory conditions^6, 7^. Patients are initially placed in a supine position on a special-purpose table, and are subsequently titled to the upright position. During all the parts of the test, continuous recording of heart rate and beat-to-beat blood pressure is performed and is subsequently analysed to determine the type of the main mechanism leading to a loss of consciousness. Spectral methods of heart rate and blood pressure variability analysis have been extensively used to describe cardiovascular rhythms and the role of the ANS activity during vasovagal syncope^8, 9^. However, the results obtained have contained numerous discrepancies^5^. Moreover, while considerable research has been devoted to ANS dysfunction assessment by HRV and BPV analysis, less attention has been paid to revealing other mechanisms responsible for syncope by using heart rate and blood pressure variability investigations.

Recently, there has been growing interest in asymmetric features of cardiovascular parameters. HRV analyses have revealed a difference in the short-term organization between heart decelerations and accelerations^10–16^. Moreover, it has been proposed that this difference could be related to the rate^17–19^, and the pattern of breathing^20^. Also by studying BPV, differences between rises and falls in systolic blood pressure have been identified^21^. Indeed, interbeat time series do not exhibit time reversal symmetry^22^ — decelerations in original time series become accelerations in the series with the inverted temporal axis/time, and vice versa. This suggests that asymmetric time patterns characterize the underlying dynamics^10, 12^, and might be used for better understanding of complex responses provoked by the HUTT.

Additionally, various methods have been proposed to assess long-range characteristics in time series data in general, and in heartbeats in particular; see, e.g., refs 22–25. In this context, an important technique used for time series assessment is based on the scaling properties of the so-called structure function differentiating between the effects of large and small fluctuations.

Here we develop an approach extending the methodology of the structure function to explicitly address asymmetry properties across the entire range of effects of large and small fluctuations. Technically, the novelty of our approach consists of splitting fluctuations into increments and decrements. Then we investigate the balance between the increments and decrements (both separately and cumulatively), from the smallest to the largest changes. In particular, the present proposition — to be referred to as a multistructure index (MI) — reveals the structure of the asymmetry between increments and decrements by studying the participation of positive increments in the pool of all changes. We hypothesize that fluctuations of various sizes might characterize distinct mechanisms of cardiovascular regulation.

Therefore, by investigating similarities and differences in MI between healthy individuals and patients suffering from VVS, in a supine position and in three separate time windows during tilting, we can observe and compare coordinated activities of basic mechanisms responsible for cardiovascular homeostasis. Remarkably, we obtain differentiation between all the groups investigated, from healthy to pathological — VVS patients, not only in the three windows of tilting, but in the actual initial condition — supine position prior to tilting. This capability of the MI index to provide precursory insight into the syncope mechanism by means of investigating time series asymmetry constitutes the main innovation of this work.

The paper is organized as follows. The Methods section contains a description of the subject data, together with a full explanation of the methodology of signal acquisition, pre-processing, and the statistical analysis used. The definition of the MI-index and an introduction to its capacity as an index of asymmetry is provided in the Methods section. The Results section presents graphical MI(q) plots of groupwise responses to tilting for both RR and SBP increments. A discussion of the results and conclusions close the manuscript.

## Materials and Methods

### Subjects and tests

The study comprised signals obtained from 112 vasovagal patients who experienced vasovagal syncope in their everyday life and 29 healthy individuals with no history of fainting. All clinical data came from the Department of Cardiology and Electrotherapy, Medical University of Gdansk, Poland. The experimental protocol complied with the Declaration of Helsinki and was approved by the Bioethics Committee of the Medical University of Gdansk. All the subjects gave their informed written consent to participating in the study.

The study group was recruited from patients managed in the outpatient syncope evaluation clinic. Inclusion criteria were: the occurrence of at least 2 episodes of syncope within the last 6 months, a typical history suggesting vasovagal fainting, and age 18–50 years. Patients with a history or physical examination findings suggesting mechanisms of syncope other than vasovagal were excluded. The control group (CG) consisted of healthy volunteers matching the study group in age and sex. None of the subjects were medicated. A demographic description of the groups studied is presented in Table 1.

**Table 1:** General description of the groups studied.

Group name:	CG	NEG	VVS1	VVS2
Size	29	34	57	21
Gender^1^ (male female)	15/14	12/22	17/40	5/16
Age^2^ (years)	24.9 ± 1.3	29.0 ± 1.3	26.2 ± 1.0	27.8 ± 1.3
BMI^3^ (kg/m^2^)	23.9 ± 0.64	23.9 ± 0.6	23.5 ± 0.5	23.2 ± 0.9
History of syncope	no	yes	yes	yes
Syncope in test	no	no	VVS1	VVS2

Data of age and BMI are presented as average values ± standard error.

^1^By Kruskal-Wallis ANOVA, there is no statistically significant difference between the groups in the gender structure (p = 0.147).

^2^By two-way ANOVA, there are no statistically significant differences across groups with respect to age (p = 0.477) or gender (p = 0.122), nor in the interaction between age and gender (p = 0.877).

^3^By two-way ANOVA, there are no statistically significant differences in BMI with respect to groups (p = 0.859), nor is there interaction between the groups and gender (p = 0.212). The difference in BMI with respect to gender is statistically significant (p < 0.001).

The HUTT tests were performed in all the study participants between 7 and 11 am, after an overnight fast. The subjects rested in a supine position on a special table for 30 min and then the table was tilted to 60°. The study participants remained in the tilted position for 20 minutes or until the occurrence of syncope. If fainting did not occur, the subject was administered 400 micrograms of nitroglycerine (aerosol, sublingually) and the test was continued for 10 minutes or until syncope occurred. During the tests, all the subjects were asked to synchronize their breathing rhythm with recorded, verbal instructions to breathe in and out at a frequency of 0.25 Hz, in the physiological breathing pattern, namely with a 1:2 ratio between the inspiration and expiration time.

The results of the HUTT test were interpreted according to the modified VASIS criteria^26^. If the blood pressure fell, followed by an increase in the RR-intervals to less than 1.5s (or if the RR-intervals increased to more than 1.5s, but this increase lasted less than 10s, so that asystole did not occur), the syncope was classified as being of mixed type and was denoted as VVS1. If the RR-intervals increased to an asystole of more than 3s (or if the occurrence of RR-intervals greater than 1.5s lasted longer than 10s, together with or before a drop in blood pressure), the syncope was described as cardioinhibitory and was denoted as VVS2 type.

As the various types (results) of vasovagal syncope are related to different pathophysiological backgrounds, we decided to analyze their data separately. Therefore, our study groups were as follows: **CG** - control group (healthy individuals); **NEG** - patients with a history of syncope who did not faint during HUTT; **VVS1** - patients with a history of syncope who experienced mixed type vasovagal syncope during HUTT; **VVS2** - patients with a history of syncope who experienced faint of cardioinhibitory type during the test.

### Signal acquisition

Initially, the study group consisted of 50 healthy subjects, 100 patients with a positive result for the HUTT test and 50 patients with negative result for HUTT (no syncope during the test). Subjects who fainted before nitroglycerine administration were not included in the present study (8 patients). Additionally, 14 healthy subjects who experienced syncope during HUTT (despite having no history of syncope) were also excluded from our study.

Surface ECG (lead II) and beat-to-beat pressure were simultaneously recorded using the Task Force Monitor System (CNSystems Medizintechnik GmbH, Austria). Data were sampled at 1 kHz. All the patients were examined in the same room at the same temperature. The Task Force Monitor System is a device for continuous noninvasive assessment of cardiovascular regulation. (The Task Force Monitor System is particularly useful in the diagnostic processing of patients with syncope. With the special software, ECG and blood pressure are recorded and visualized during the HUTT test and then all the data are ready for export. Blood pressure is measured beat-to-beat on the finger artery with a double finger sensor, using CNAP technology. With the so-called vascular unloading technique, plethysmographic signals are transformed into continuous blood pressure information. The so-called VERIFI-algorithm is applied to overcome the problem of bias caused by vasoconstriction or vasodilatation).

Despite all the attempts to obtain signals of the best quality, in 7 subjects in the control group and 30 vasovagal patients, the ECG and/or blood pressure recording was not satisfactory to obtain reliable results (if the artefacts in a signal involved in total more than 5% of values then the patient’s recording was excluded from the further analysis; signals with less than 5% artefacts were edited — the erroneous values were replaced by the median of the surrounding seven correct values) and we had to exclude these individuals from the groups studied. As a result, the present paper is based on data from 112 patients and 29 healthy volunteers.

### Signal pre-processing

The values of RR-intervals and SBP levels will be denoted, respectively, as *RR*_*i*_ and *SBP*_*i*_, where *i* indexes subsequent normal heart contraction events. Then RR-increments are defined as changes in the length between the adjacent RR-intervals, i.e., ${\rm{\Delta }}R{R}_{i}:=R{R}_{i}-R{R}_{i-1}$, and SBP-increments as changes in SBP between the subsequent beats, i.e., ${\rm{\Delta }}SB{P}_{i}:=SB{P}_{i}-SB{P}_{i-1}$.

**Figure 1:**
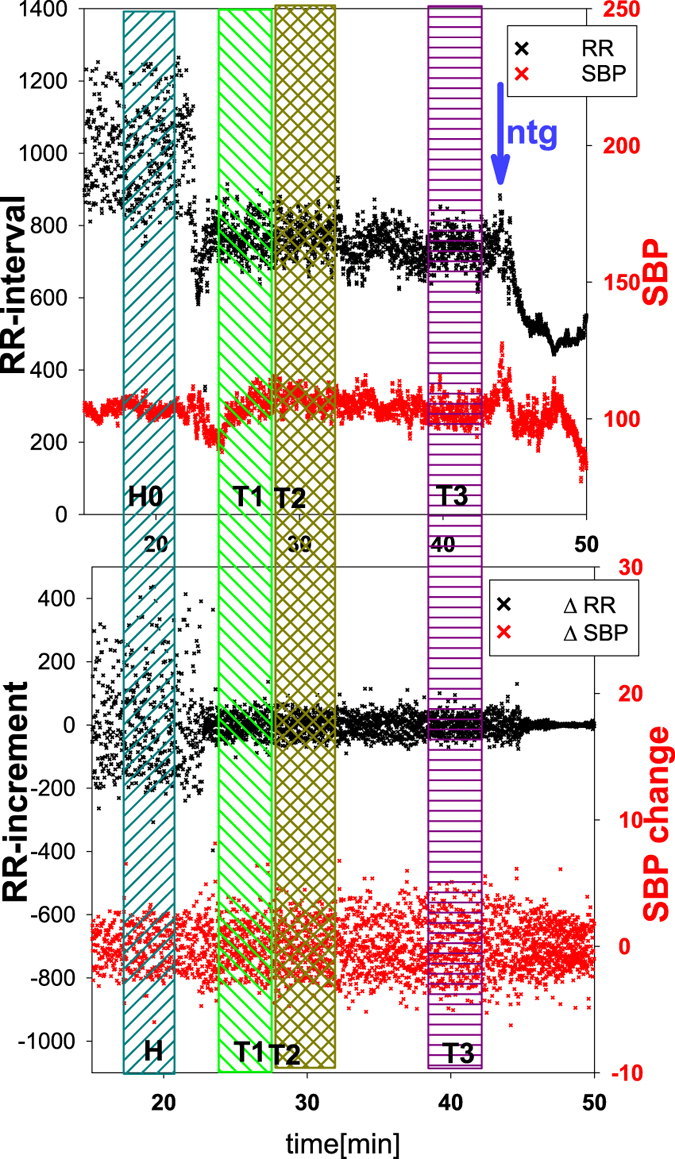
A typical recording of RR-intervals (in ms) and SBP (in mmHg) and signals with RR-increments and SBP-increments obtained during the HUTT test for a subject who fainted after nitroglycerine (NG) administration. Each recording was analyzed in four time windows denoted as H, T1, T2 and T3, consisting of 300 beats each.

In Fig. 1, top, a typical recording of RR-intervals and values of SBP are shown. These signals were obtained from a subject who fainted (VVS1 type) in the HUTT test. In the bottom part of the figure, the changes in RR-intervals and in SBP are plotted.

The procedure of tilting results in nonstationarity of the signals. In order to facilitate the investigation of the dynamical effects after the onset of tilting, each signal was divided into windows consisting of 300 beats. The first window (named H for horizontal) contains recordings which ended one minute before tilting, when the subject was still in the supine position. Subsequently, one minute after tilting, two windows of 300 beats were selected, to be referred to as an early tilt phase T1 (for term 1, etc.), and the actual tilt phase T2. The final, late tilt phase window of 300 beats, referred to as T3, entails beats recorded one minute before nitroglycerine administration.

### Multistructure index (MI): definition

Let us denote as Δ(*i*) a change between the adjacent *i* and *i* − 1 values of either the RR-intervals or the values of SBP. Moreover, let ${{\rm{\Delta }}}^{+}(i):={\rm{\Delta }}(i) > 0$ denote a positive change in the variable, and ${{\rm{\Delta }}}^{\ne 0}(i):={\rm{\Delta }}(i)\ne 0$ denote any change different from zero. Then, for any real number *q*, the MI is defined as follows:

1 $$MI(q):=\frac{{\sum }_{{{\rm{\Delta }}}^{+}(i)}{({{\rm{\Delta }}}^{+}(i))}^{q}}{{\sum }_{{{\rm{\Delta }}}^{\ne 0}(i)}{|{{\rm{\Delta }}}^{\ne 0}(i)|}^{q}.}$$

The function *MI*(*q*) measures the total effect of the positive changes with respect to all changes in the signal, when the influence of the changes of a particular size is selectively magnified (enhanced or suppressed) by the particular value of the control parameter *q*. Note, that if for each size of the change there is a balance between positive and negative changes then *MI*(*q*) = 0.5 independently of *q*. However, if there are event sizes where prevalence or deficiency of Δ^+^s occurs, then depending on the size of Δ^+^, small/mid-size/large, values different from 0.5 occur at different intervals of *q*, namely for negative/around 0/positive values of *q*, respectively.

### *MI*(*q*) as an index of asymmetry

Let *MI*_RR_(*q*) refer to the multistructure index of RR-increments. Then *MI*_RR_(*q*) estimates the proportion of decelerations Δ^+^s in all changes of the heart rhythm. Similarly, let *MI*_SBP_(*q*) refer to the multistructure index of SBP-increments. Then *MI*_SBP_(*q*) measures the role of rises in SBP Δ^+^s with respect to all non-zero SBP-increments.

From the mathematical point of view, the smallest and biggest increments in a signal function as anchors stabilizing the limit values of *MI*(*q*). This implies that the value of *MI*(*q*) for large negative *q* is determined by whether or not the smallest positive and negative values are balanced. Similarly, the ratio between positive and negative the biggest values determines *MI*(*q*) for large *q*. Similar proportions for other signal values influence the q-spectrum of *MI*(*q*) when *q* moves from large negative to large positive values. However, the relation between small, mid-size and large events is logarithmic, which implies that with this distinction, we separate dynamics of different timescales.

In the following, we consider the properties of *MI*(*q*) observed for *q* < −2 as referring to the ‘smallest changes’, and for *q* > 2 as referring to the ‘largest changes’ in the signal. The features obtained for −2 ≤ *q* ≤ 2 will be considered as ‘mid-size events’. Therefore, the smallest changes can be interpreted as referring to the slow dynamics, and the largest changes as describing the fast dynamics. Note that in the case of RR-intervals and SBP signals, the probability of a change sharply decreases with the increase in the size of the event. Therefore, the smallest changes are the most probable changes, whilst the largest changes correspond to ‘rare events’.

In contrast, non-negative and integer *q* values can be related to the moments of the probability distribution of increments. Accordingly,

- If *q* = 0, then *MI* is the ratio of the total number of positive changes of any size to the total number of all changes. Hence, *MI*_RR_(0) answers the question about which events happen more frequently, decelerations or accelerations. Similarly, *MI*_SBP_(0) tells whether falls or rises in SBP are more frequent.
- If *q* = 1, then *MI* provides the ratio of the total sum of Δ^+^s to the total sum of all changes. In this way, *MI*(1) provides information about whether there is a trend in the signal or not, whether the values of the signal, RR-intervals or SBP levels are increasing or decreasing. *MI*_RR_(1) is directly related to the Porta index *PI* ^12^, as *MI*(1) = 1 − *PI*.
- If *q* = 2, then *MI* estimates the ratio of fluctuations of Δ^+^s to the total of all fluctuations in the signal. Therefore *MI*(2) provides information about the overall variability of Δ^+^s with respect to the variability of the signal. *MI*_RR_(2) is known as the Guzik index *GI* ^11^, *MI*_RR_(2) = *GI*.
- If *q* = 3, the *MI* explores the skewness of the probability distribution of increments. In this way, *MI*(3) can provide characterization equivalent to the skewness index *S*_1_ considered in ref. 13.

### Numeric and statistics estimates

The *MI*_RR_(*q*) and *MI*_SBP_(*q*) functions were calculated for each signal with RR-intervals and with values of SBP, respectively, in each time window separately, for $q\in [-5,5]$ with step Δ*q* = 0.1. Then by pooling the results corresponding to the same group of subjects and the same time window, we obtained the group mean ${\overline{MI}}_{{\rm{RR}}}(q)$ for the given set of RR-intervals, and ${\overline{MI}}_{{\rm{SBP}}}(q)$ for SBP.

Formula (1) makes it possible selectively to amplify singular unbalanced extremes – minimal and maximal changes in the signal. However, minimal changes are usually related to the properties of the equipment used rather than to the physiological properties recorded. Also maximal changes may be more related to individual characteristics of a subject than representative of the features of the group. Therefore our calculations were performed under the following restrictions to extremal values:

$$\begin{matrix}\quad \quad \,\,3\,{\rm{ms}} & \le  & {\rm{\Delta }}R{R}_{i}\le 200\,{\rm{ms}}\,{\rm{for}}\,{\rm{H}}\\ \quad \quad \,\,3\,{\rm{ms}} & \le  & {\rm{\Delta }}R{R}_{i}\le 100\,{\rm{ms}}\,{\rm{for}}\,{\rm{T}}1,{\rm{T}}2,{\rm{T}}3\\ 0.2\,{\rm{mmHg}} & \le  & {\rm{\Delta }}SB{P}_{i}\le 10\,{\rm{mmHg}}.\end{matrix}$$

Applying these thresholding criteria, the spectrum of quantified events spreads over three clearly separated logarithmic timescales. Estimates performed with different limits potentially modify the significance of the smallest, and largest events. The effect might potentially also affect the properties of mid-size events, which could, for example, be less pronounced since the specific features would accumulate in the mid-size events due to noise from extreme values. With these study limitations in mind, we nevertheless consider the thresholding criteria applied to be reasonably conservative.

The numerical and statistical validation of the group means was performed with MATLAB and its Statistics Toolbox R2016b (The MathWorks Inc.). The overall effect of the four factors (groups of patients, time windows, exponential index *q*, type of the signal) on the mean of the function *MI* was studied by four-way analysis of variance. The interactions among pairs of factors were taken into account. Analysis was performed for different sets of *q*, starting from classical {1, 2} to represent the Porta and Guzik indices alone. Based on the *anovan*() results, multiple comparisons (*multicompare*()) were performed for pairwise comparison of the group means.

We applied the Lilliefors test to assess the normality for each *q* value of the pooled group results for each *q*. Since the majority of the results passed the normality test (at significance level *p* < 0.05), we represent the group results as their means and standard errors of the means. The asymmetry of MI, i.e., the validity of the distinction of the *MI*(*q*) value from 0.5 was tested by two-sided *t*-test. The statistical significance of the differences between two groups was verified by *t*-test for either paired data (in the case of the same group of patients) or independent data (in the case of different groups of patients). The figures were prepared with SigmaPlot V13.0 (Systat Software Inc.).

## Results

If *q* = 1, 2, the *MI* response to each factor (groups of patients, time windows, index *q*, type of the signal) of the statistical analysis is proven to be statistically significant with *p*-values (0.0005, 0.0033, 0, 0), respectively. However, only the interaction of the time window and *q* index has had a statistically significant (*p* = 0.0384) impact on the mean of *MI*. By adding −0.5, 0, 0.5 to the set of *q* values, we obtained not only better statistics for the impact of all the groups [*p*-value: (0, 0.0223, 0, 0)] but also all the pairs of interactions were statistically significant with the exception of the pair (groups of patients, exponent *q*). A statistically significant impact of this pair was obtained when large *q* negative values were considered. For example, for *q* = −5.0, −0.5, −0.2, 0.2, 0.5, 1.0, 1.5, 2.0 all factors and also all pair interactions reached the *p*-value less than 0.05.

**Figure 2:**
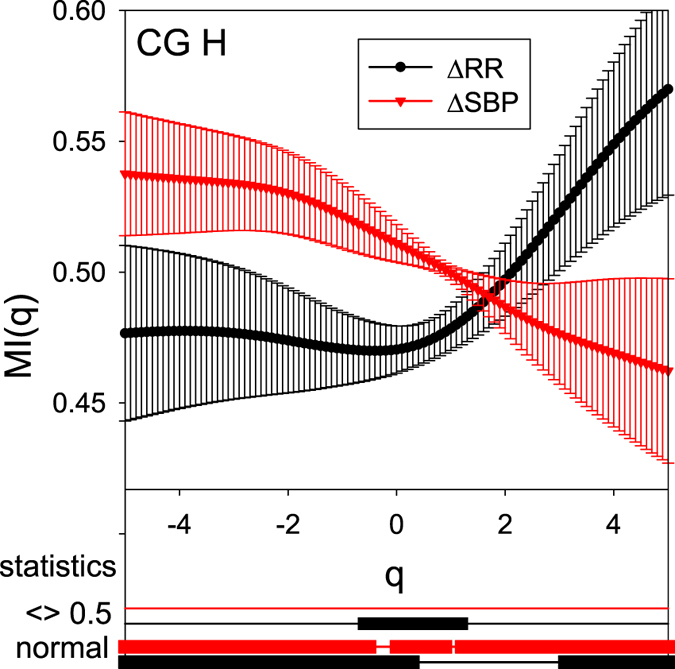
The mean ${\overline{MI}}_{{\rm{RR}}}(q)$ and ${\overline{MI}}_{{\rm{SBP}}}(q)$ (with std errs) obtained from the signals of the CG group in the supine position H. Below the *q*-axis, the statistics of the group results are given subsequently: bottom two lines mark *q* intervals in which the hypothesis of normality cannot be rejected (Lilliefors test, *p* < 0.05); top two lines mark *q* intervals in which *MI*s are distinct from 0.5 (two-sided *t*, *p* < 0.05). Black curves refer to RR data; red curves describe SBP data.

**Figure 3:**
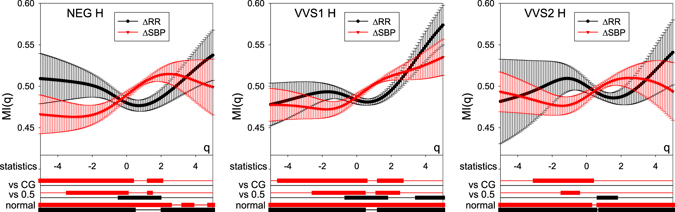
Plots of the mean ${\overline{MI}}_{{\rm{RR}}}(q)$ and ${\overline{MI}}_{{\rm{SBP}}}(q)$ (with std errs) obtained for patient groups NEG, VVS1 and VVS2 in the time window H. Below the *q*-axis, the statistics of the group results are given subsequently: bottom two lines mark *q* intervals in which the hypothesis of normality cannot be rejected (Lilliefors test, *p* < 0.05); middle two lines mark *q* intervals in which *MI*s are distinct from 0.5 (two-sided *t*, *p* < 0.05); top two lines mark *q* intervals in which *MI*s are distinct from the corresponding *MI*s of the CG (*t*-test for independent data, *p* < 0.05). Black curves refer to RR data; red curves describe SBP data.

**Figure 4:**
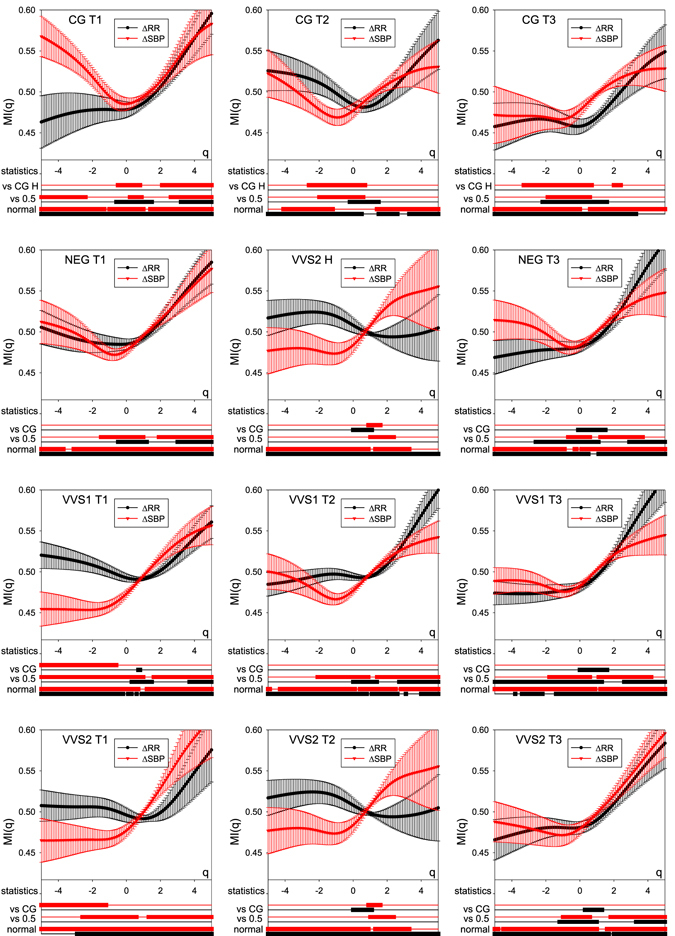
The means ${\overline{MI}}_{{\rm{RR}}}(q)$ and ${\overline{MI}}_{{\rm{SBP}}}(q)$ and their standard errors obtained for all groups considered in time windows after tilting. Below the *q*-axis, the statistics of the group results are given subsequently: bottom two lines mark *q* intervals in which the hypothesis of normality cannot be rejected (Lilliefors test, *p* < 0.05); middle two lines mark *q* intervals in which *MI*s are distinct from 0.5 (two-sided *t*, *p* < 0.05); top two lines mark *q* intervals in which *MI*s are distinct from the corresponding *MI*s of the CG (*t*-test for independent data, *p* < 0.05). Black curves refer to RR data; red curves describe SBP data.

The dependence of ${\overline{MI}}_{{\rm{RR}}}$ and ${\overline{MI}}_{{\rm{SBP}}}$ on *q* for all the groups of the signals studied are jointly plotted in Figs 2, 3 and 4. Together with these plots the crucial statistical properties are presented in graphical form below the MI plots. In particular, beginning from the bottom of each figure, the solid lines describe the *q* values for which the normality test has been passed (thick line) — which validates our use of the means in presenting group properties. Then, the next lines up show the ‘significance’ (thick line) of the detection of asymmetry — i.e. the test if the actual *MI*(*q*) value is significantly distinct from 0.5. Finally, the top lines refer to the significance of the difference with respect to the results obtained for the CG group. The results of this test support our investigations of pathological cardiovascular regulation of homeostasis in VVS patients.

### The baseline

Plots of ${\overline{MI}}_{{\rm{RR}}}(q)$ and ${\overline{MI}}_{{\rm{SBP}}}(q)$ obtained for the supine position, shown in Figs 2 and 3, present the baseline properties of the dynamical organization in the homeostatic state at rest in healthy people and in vasovagal patients.

For small and mid-size increments, i.e. for *q* < 2, both the indices ${\overline{MI}}_{{\rm{RR}}}(q)$ and ${\overline{MI}}_{{\rm{SBP}}}(q)$ remain in the vicinity of and do not substantially depart from the value 0.5, which corresponds to balanced participation of negative and positive increments (see Fig. 2). However, the domination found for SBP signals of Δ^+^ is distinct from the domination observed for the RR-intervals, Δ^−^. Additionally, there is a noticeable transition in the predominance in both signals, SBP and RR-intervals, when *q* is changing from the negative *q* values to positive *q* values. These two properties together make the plots of the indices ${\overline{MI}}_{{\rm{RR}}}(q)$ and ${\overline{MI}}_{{\rm{SBP}}}(q)$ appear symmetrical with respect to the line *MI*(*q*) = 0.5 and with respect to the point (0, 0.5) when compared with each other.

We observe the slight domination among the most probable events, i.e., accelerations in the case of RR-intervals and rises in the case of SBP, related to the events of the smallest size. The prevalence of rare events is likely to be related to large decelerations in the heart rate and large falls in the blood pressure. It is worth noting that the domination of accelerations extends to all RR-increments except the largest RR-increments, because ${\overline{MI}}_{{\rm{RR}}}(q)$ is lower than 0.5 for *q* < 2 (approximately).

The functions ${\overline{MI}}_{{\rm{RR}}}(q)$ and ${\overline{MI}}_{{\rm{SBP}}}(q)$, shown in Fig. 3 are substantially different from the functions found in the group of healthy people presented in Fig. 2. These differences can be enumerated as follows:

- Only the indices of the NEG group are mutually symmetric and along the line *MI*(*q*) = 0.5, but there is no symmetry with respect to the point (0, 0.5). The indices of the VVS1 and VVS2 groups are not mutually symmetrical either along the line *MI*(*q*) = 0.5 or at the point (0, 0.5).
- There is an evident distinction in the prevalence of the most probable events for SBP between healthy people and vasovagal patients. In vasovagal patients, the most probable events, the smallest ones, exhibit the domination of falls in SBP, while in the case of the healthy people, rises in SBP dominate over falls. This distinction among the smallest events and in some of the mid-size events is statistically significant.
- In the case of RR-interval dynamics, for all groups there is a slight domination of accelerations among the mid-size RR-increments. Considering large changes, the signals from the healthy group display a significant prevalence of decelerations, while the signals from the vasovagal groups do not show such a strong prevalence. However, the values of ${\overline{MI}}_{{\rm{RR}}}(q)$ of the vasovagal patient groups are not significantly different from the healthy group for any *q* considered.

### The tilting

The results for the mean values ${\overline{MI}}_{{\rm{RR}}}(q)$ and ${\overline{MI}}_{{\rm{SBP}}}(q)$ obtained in response to the tilt for signals recorded from participants in the four groups considered in windows T1, T2 and T3 are shown in Fig. 4.

Note, that each plot shown in Fig. 4 is different from all the others and different from the plots presented in Figs 2 and 3. In particular:

- In time window **T1** (the first column of Fig. 4), rises in SBP and decelerations in heart rate dominate among the largest size events. However, only for the healthy people do we see this domination among the smallest size changes. Moreover, the vasovagal patients who fainted during the test, the groups VVS1 and VVS2, demonstrate the statistically significant domination of falls. There is also a noticeable prevalence of accelerations of the mid-size in the groups of people who did not faint, CG and NEG. This prevalence in the case of the healthy people extends to the smallest size accelerations, but is not present in the NEG group.
- In time window **T2** (the second column of Fig. 4), similarly to in the T1 window, large rises in SBP dominate over large falls in all the groups. However, the more frequent events of the mid-size exhibit a domination of falls in SBP in all groups. The mid-size changes in RR-intervals show a slight prevalence of accelerations in all groups, although in the case of the VVS patients, this feature is only weakly manifested in the cases of NEG and VVS1, and even absent, in the case of VVS2.
- Finally, in time window **T3** (the third column of Fig. 4), in the late tilt window, in all the groups there is a prevalence of rises in SBP among the largest events. There is no significant domination among other events. Despite the domination of decelerations between the largest RR-increments, the heart rhythms show an evident prevalence of the mid-size and smallest accelerations in all the groups.

The functions ${\overline{MI}}_{{\rm{RR}}}(q)$ and ${\overline{MI}}_{{\rm{SBP}}}(q)$ estimated from signals of controls define the healthy physiological response to the tilting challenge. In the case of SBP, this response relies on the switch in domination of large changes in SBP: from the prevalence of falls in the supine position to the prevalence of rises after tilting, which occurs in all time windows; see the first row in Fig. 4. Also among the smallest SBP-increments, we see a slight domination of rises. This domination, however, decays with time, turning into a domination of falls in the T3 window. Considering the heart rate, similarly to the supine state, the largest decelerations are more frequent than the largest accelerations. Surprisingly, as time passes after titling, a domination among the smallest RR-increments oscillates from a slight domination of accelerations in the early tilt time window T1 to the prevalence of decelerations in T2, and back to the domination of accelerations in T3.

The properties of ${\overline{MI}}_{{\rm{RR}}}(q)$ and ${\overline{MI}}_{{\rm{SBP}}}(q)$ obtained from vasovagal patients are distinct from the above-listed fingerprints of the healthy response. First of all, the NEG group differs in prevalence among the smallest SBP events in all time windows (though, the difference is not statistically significant). Moreover, the domination of mid-size accelerations in T3 is not as strong as in the CG. The VVS1 group differs in the prevalence of falls among the smallest SBP increments in T1. The domination of mid-size accelerations is not as evident as in the CG. The VVS2 group differs in the prevalence of falls among the smallest SBP increments in T1. Here in the T2 window, we see that the largest decelerations are balanced by accelerations of a similar size.

Summarizing the above, the prevalence of falls among the smallest SBP-increments in the early tilt window T1 is a distinctive feature of vasovagal patients who fainted during the test, i.e., the groups VVS1 and VVS2.

## Discussion

Our proposition of MI for quantification of events according to their sizes and directions follows a popular approach of the structure function used in the estimates of long-range dependencies in time series {*x*_*i*_}, i.e. $\langle {|{x}_{i+m}-{x}_{i}|}^{q}\rangle$, where the average 〈〉 is taken over all possible pairs (*x*_*i*+*m*_, *x*_*i*_) separated by *m* steps in time. Negative and positive *q* values respectively emphasize the collective effect of small and large fluctuations in *x*_*i*_ values. In ref. 16, we applied this kind of technique to explore the asymmetry between accelerations and decelerations in beat-to-beat subsequent heart contractions via differences between distributions of accelerations and decelerations. The Generalized Porta Index (GPI) was proposed, with which we hypothesized that it should be possible to distinguish healthy people from VVS patients based on signals before the HUTT test. MI might appear, at first, mathematically similar to GPI, however, it relies on a substantially different concept. While the GPI quantifies asymmetry based on the probability of a given change, the MI refers to asymmetry with respect to the size of the change. What follows is the ability to contrast properties of slow dynamics (small changes that could be related to tonic activity of sympathetic and parasympathetic systems) with fast dynamics (large changes that might be assumed to result from activation of feedback loops of autonomic responses to the actual state of the organism), which could be directly related to known physiological processes.

However, the influence of these relationships is assessed following a multiscaling approach, meaning that the results do not necessarily progress smoothly from the smallest to the biggest values, but rather tend to exhibit strong non-linear increases/decreases over scales. Hence, by the suitable choice of the minimal and maximal increments we can directly not only identify, but also separate the scales of interest, such as, for example, accelerations and decelerations larger than 50 ms commonly associated with vagal activity, from smaller ones which are usually considered as following the sympathetic activation.

During the study, the subjects were initially in supine rest. Head-up tilting then triggered the autonomic provocation. Our aim was to characterize the dynamics of the short-term regulation of the two basic physiological rhythms: changes in RR-intervals and changes in SBP. It is commonly believed that RR-intervals and the values of SBP are entangled with each other, which leads to beat-to-beat variations in the length of RR-interval and in the value of SBP. The so-called feed-forward mechanical pathway (Starling law and diastolic runoff) is responsible for the effect of the actual RR-interval on SBP, and baroreflex feedback induces the variation in RR-intervals in response to SBP change. The baroreflex loop is assumed to be crucial in maintaining the proper blood perfusion in distant organs. Investigation of baroreflex effects demands at least bi-variate signals. Our signals with RR and SBP were indeed bi-variate signals, but we estimated properties of RR and SBP separately. However, in a rough approximation, the baroreflex sensitivity assumes linear dependence between Δ*RR* and Δ*SBP*. Therefore, baroreflex couplings can be qualified by symmetries between asymmetries in ${\overline{MI}}_{{\rm{RR}}}(q)$ and ${\overline{MI}}_{{\rm{SBP}}}(q)$ ^27^. Note, that such an approach avoids the problem of delays between RR-interval and SBP beat-to-beat interactions.

It is known that beat-to-beat patterns are strongly influenced by respiration^9, 18, 28, 29^. Typically, heartbeats are shortened (heart rate accelerates) during inspiration, and prolonged (heart rate decelerates) during expiration. However, the exact phase relationship happens to be dependent on the respiration rate^18, 20, 30^. Therefore, a paced breathing protocol at frequency 0.25 Hz was applied to our subjects with the physiological ratio of 1:2 inspiration to expiration. Although such breathing results in breaths deeper and more rapid than spontaneous breathing, it is considered best to resemble normal breathing^17^.

Under the baroreflex hypothesis of heart rate and blood pressure interactions, rises in SBP cause a deceleration of the heart rate, and falls in SBP result in an acceleration of the heart rate. This schema can be directly read from the MI’s of healthy people at rest, where slow, mid and fast dynamics of RR-increments and SBP are symmetrical to each other. However this picture is not as clear in the other groups considered. Let us explain the differences below.

It is commonly believed that at rest, we should observe effects of the sympathetic tonic activity pacified by increased vagal activity. From *MI*(*q*) estimates, we see that in healthy people at rest, the slow dynamics (the small and mid-size changes), which could be attributed to the tonic activity of the sympathetic nerves, is likely to rely on small accelerations and SBP rises, which are punctuated by greater decelerations and large falls, respectively. Therefore, we can hypothesize that the tonic sympathetic activity in vessels results in an increase in the blood pressure, which engages the baroreceptor reflex, activating the vagal system to slow down the heart rate.

Yet, the signals of vasovagal patients provide a different picture of the homeostatic regulation. The dynamical pattern of SBP increments is opposite or different to the regulation found in healthy people. Namely, there is a slight prevalence of small SBP falls over all small events. In the case of the NEG group, this is accompanied by a slight domination of small size decelerations. This could suggest a weak and potentially insufficient effect of tonic sympathetic activity to vessels that should activate the baroreflex driven intervention to accelerate the heart, hence to withdraw vagal activity. However, we observe the prevalence of accelerations only among mid-size events and not among the largest ones. The Porta index, $PI=1-{\overline{MI}}_{{\rm{RR}}}\mathrm{(1)}$, in all groups in the H window exhibits a small prevalence of accelerations over decelerations. The Guzik index, ${\overline{MI}}_{{\rm{RR}}}\mathrm{(2)}$, also shows such a systematic prevalence of accelerations, probably because *q* = 2 is close to the border between the mid-size and largest RR-increments. The presence of these large decelerations could be thought of as a manifestation of the strong parasympathetic activation caused by the driven (i.e. paced) expiration. Thus, the homeostatic state in vagal sensitive patients appears to be maintained by two opposing signals sent to the vagal nerves: to speed up the heart (sent from the vascular system), and to slow down the heart (sent from the respiratory system).

Following the above observations, a very rough description of the underlying dynamics — ‘zero approximation’ can be suggested as follows. Let us assume that slow and fast dynamics correspond to distinct regulatory mechanisms coexisting together, which, however, are switched on-off in some specific manner. Then heart period *RR* and systolic blood pressure *SBP* change from beat to beat in the following way:

2 $${\rm{\Delta }}RR(i)={\xi }_{n}^{RR}(i)-{\delta }^{RR}(i){p}_{s}^{RR}+{{\rm{\Delta }}}^{RR}(i){p}_{f}^{RR}$$

3 $${\rm{\Delta }}SBP(i)={\xi }_{n}^{SBP}(i)+{\delta }^{SBP}(i){p}_{s}^{SBP}-{{\rm{\Delta }}}^{SBP}(i){p}_{f}^{SBP},$$

where ${\xi }_{n}^{RR}$, ${\xi }_{n}^{SBP}$ are noise type variables with zero mean for the smallest changes; *δ* ^*RR*^, *δ* ^*SBP*^ stochastic variables centred at some non-zero values which describe slow dynamics; ${p}_{s}^{RR}$, ${p}_{s}^{SBP}$ on-off probability of activation of slow dynamics; Δ^*RR*^, Δ^*SBP*^ stochastic variables centred at some non-zero values describing fast dynamics; ${p}_{f}^{RR}$, ${p}_{f}^{SBP}$ on-off probability of activation of fast dynamics. In the case of healthy people, we have 0 < *δ* ^*SBP*^ < Δ^*SBP*^, while in the case of VVS patients, the relation seems to be opposite, i.e., 0 > *δ* ^*SBP*^ > Δ^*SBP*^.

To our knowledge, *MI*(*q*) is the first index which so clearly distinguishes cardiovascular autonomic regulation of healthy people from vasovagal patients based on recordings at rest. MI identifies the opposite roles of the basic mechanisms maintaining the homeostasis.

The tilt provocation leads to a switch in the fast dynamics of SBP. Large rises in SBP are observed in all the groups in all time windows, which could be the effect of strong sympathetic stimulation. However, since we still observe an excess of occurrences of the largest heart decelerations, we might suppose that the heart rhythm remains under the strong influence of respiratory action. It is noticeable that only in healthy people are these rapid changes in SBP supported by slow dynamics, earlier attributed to tonic sympathetic activity, and that this support vanishes with time after tilting in healthy people, whilst in the case of vasovagal sensitive patients, it seems to increase. Therefore, we hypothesize that changes in the tonic sympathetic response, probably driven by adrenergic processes, could be slow or inefficient in the case of vasovagal patients. The effect is most pronounced in the early tilt window.

Under normal physiological conditions, distribution of the blood is regulated by vascular resistance^1, 31^. The resistance is determined by the diameters of precapillary vessels, which are normally partially constricted. This state, called vascular tone, is maintained by smooth vascular muscle cells, which are located in the wall of the vessel. There are many vasoactive substances produced by the tissue cells around the resistance vessels. Vascular tone emerges as the net effect of vasoconstrictor and vasodilator influences acting on the vessel. The factors which determine the degree of smooth muscle activation are traditionally divided into the extrinsic: sympathetic nerves, circulating hormones, and the intrinsic: endothelial-derived factors, smooth muscle myogenic tone, locally produced hormones, and tissue metabolities^1^. Some of these factors promote vasocontriction (e.g., sympathetic nerve, angiotensin II, and endothelin-1, myogenic), whereas others promote smooth muscle relaxation (nitric oxide derived from endothelium and tissue metabolites such as adenosine and hydrogen ion).

At rest, vascular tone is maintained locally. The extrinsic neurohumoral influence related to the baroreflex is low, and the intrinsic mechanisms of the blood vessels – myogenic tone and the tissue metabolism surrounding blood vessels – dominate. Our results suggest two different ways in which the vascular tone is preserved in healthy people and in vasovagal patients. At rest, a homeostatic state of SBP in healthy people is maintained by smaller size rises, which are balanced by falls of a larger size. In the case of vasovagal patients, we find the opposite result: small falls are balanced by larger rises. In analogy to heart period indices, we can consider the Porta index for SBP to be $1-{\overline{MI}}_{{\rm{SBP}}}(\phantom{\rule{-0.25em}{0ex}}+\phantom{\rule{-0.25em}{0ex}}\mathrm{1)}$ and the Guzik index, ${\overline{MI}}_{{\rm{SBP}}}(\phantom{\rule{-0.25em}{0ex}}+\phantom{\rule{-0.25em}{0ex}}\mathrm{2)}$. These indices, especially the Guzik index, capture and characterize the distinct role of rises. Hence in healthy people, we see that the activity of contractors is compensated for by vasodilators, but in vasovagal patients the mechanisms of vasodilation seem to dominate over contractions. Since nitric oxide release from the endothelium might be considered to be the principal mechanism for regulating blood pressure^32^, we can speculate that our observation reveals endothelium dysfunction.

Under autonomic provocation, when the tilt test switches the autonomous nervous system regulation via stimulation of the baroreceptors, the main disparity from the resting state occurs in the properties of large rises in SBP. In all the groups studied and all the time windows, we observe a domination of these fast rises over falls. This might denote the strong activation of the mechanisms of the vessel contractions, namely strong sympathetic activity. Together with the prevalence of fast decelerations, which has been observed in all groups and time windows with the exception of the VVS2 group in T2, this suggests that the parasympathetic part of autonomic nervous system also remains activated.

### Method limitations

The MI approach has two sources of limitations. First of all syncope is a phenomenon which is characterized by transient properties in *RR*-interval and *SBP* dynamics. Our analysis performed in time windows allowed us only to discern such transients which persist for longer time intervals. Transients taking short time periods might be not discovered using the window-based method. Secondly, baroreflex entanglement of heart period and blood pressure, when represented and investigated through relationships, correspondence and symmetries between two univariable functions of ${\overline{MI}}_{{\rm{RR}}}(q)$ and ${\overline{MI}}_{{\rm{SBP}}}(q)$ provides only a qualitative approximation of the phenomena. Furthermore, the cause-effect analysis between RR-intervals and SBP potentially strongly overestimates RR-intervals and SBP interactions when respiratory rhythm is not taken into account^29^. The sizes of the groups studied are modest, which implies that statistical significance was not always achieved. Finally, we used well-motivated thresholds for RR-increments and SBP-increments, which need to be included in considerations of the results.

## Conclusion

The proposed multistructure indices ${\overline{MI}}_{{\rm{RR}}}(q)$ and ${\overline{MI}}_{{\rm{SBP}}}(q)$, with *q* changing from negative to positive values, exhaustively scan the distributions of RR-increments and SBP-increments for changes of specific size. The prevalences are attributed to and change systematically while moving with the *q* value. Thus, they provide a full overview of the non-linear properties of these indices and an additional interpretation of the Porta and Guzik indices. They can be considered as a generalisation of these specific indices by providing insight into the other parts of the q-spectral distributions.

However, MI is a tool capable of assessing the entire multiscale size structure of the changes. Therefore, we suggest exploring the entire spectrum of the dynamics involved, revealing their non-linear dependence and irreversible character. Indeed, the methodology of the multistructure index is capable of contrasting properties of slow dynamics and fast dynamics, which could potentially be applicable to a wide range of problems where complex dynamical interactions are at play.

MI has been proved to provide insightful measures of the cardiovascular interactions provoked by the HUTT test. Differences have been found in the organization of the homeostatic state between healthy people and vasovagal patients. Specifically, it has appeared that vasovagal syncope might be related to the delayed reaction of the slow SBP dynamics. These observations might be considered as precursory signs of vasovagal sensitivity, and hence of vasovagal syncope.

## References

[1] Klabunde, R. E. *Cardiovascular Physiology Concepts* (Lippincott Williams & Wilkins, Wolters Kluwer, 2012).

[2] Jánig, W. *Integrative Action of the Autonomic Nervous System*. *Neurobiology of Homeostasis* (Cambridge University Press, 2008).

[3] TaskForce. Task force of the European Society of Cardiology the North American Society of Pacing. Heart rate variability: standards of measurement, physiological interpretation, and clinical use. *Circulation* **93**, 1043–1065, doi:10.1161/01.CIR.93.5.1043 (1996). 8598068

[4] Parati G, Saul JP, Di Rienzo M, Mancia G (1995). Spectral analysis of blood pressure and heart rate variability in evaluating cardiovascular regulation: A critical appraisal. Hypertension.

[5] Mosqueda-Garcia R, Furlan R, Tank J, Fernandez-Violante R (2000). The elusive pathophysiology of neurally mediated syncope. Circulation.

[6] Benditt, D., Sakaguchi, S., Lu, F. & Sutton, R. Head-up tilt testing. In Zipes, D. & Jalife, J. (eds) *Cardiac Electrophysiology*: *from Cell to Bedside*, 859 (Saunders/Elsevier, 2009).

[7] Calkins, H. Syncope. In Zipes, D. & Jalife, J. (eds) *Cardiac Electrophysiology*: *from Cell to Bedside*: *Expert Consult* - *Online and Print*, 913 (Saunders/Elsevier, 2009).

[8] Furlan, R., Montano, N. & Porta, A. Cardiovascular rhythms in vasovagal syncope. In Alboni, P. & Furlan, R. (eds) *Vasovagal Syncope*, 83–93 (Springer International Publishing, 2015).

[9] Porta A (2002). Quantifying the strength of the linear causal coupling in closed loop interacting cardiovascular variability signals. Biological Cybernetics.

[10] Costa M, Goldberger AL, Peng C-K (2005). Broken asymmetry of the human heartbeat: Loss of time irreversibility in aging and disease. Phys. Rev. Lett..

[11] Piskorski J, Guzik P (2007). Geometry of Poincare plot of RR intervals and its asymmetry in healthy adults. Physiological Measurement.

[12] Porta A (2008). Temporal asymmetries of short-term heart period variability are linked to autonomic regulation. American Journal of Physiology - Regulatory, Integrative and Comparative Physiology.

[13] Casali KR (2008). Multiple testing strategy for the detection of temporal irreversibility in stationary time series. Phys. Rev. E.

[14] Piskorski J, Guzik P (2012). Compensatory properties of heart rate asymmetry. Journal of Electrophysiology.

[15] Makowiec D, Kaczkowska A, Wejer D, Żarczyńska-Buchowiecka M, Struzik ZR (2015). Entropic measures of complexity of short-term dynamics of nocturnal heartbeats in an aging population. Entropy.

[16] Makowiec D (2015). Generalised heart rate statistics reveal neurally mediated homeostasis transients. EPL (Europhysics Letters).

[17] Pinna GD, Maestri R, La Rovere MT, Gobbi E, Fanfulla F (2006). Effect of paced breathing on ventilatory and cardiovascular variability parameters during short-term investigations of autonomic function. American Journal of Physiology - Heart and Circulatory Physiology.

[18] Grossman P, Taylor EW (2007). Toward understanding respiratory sinus arrhythmia: Relations to cardiac vagal tone, evolution and biobehavioral functions. Biological Psychology.

[19] Kobayashi H (2009). Does paced breathing improve the reproducibility of heart rate variability measurements?. Journal of Physiological Anthropology.

[20] Klintworth A, Ajtay Z, Paljunite A, Szabados S, Hejjel L (2012). Heart rate asymmetry follows the inspiration/expiration ratio in healthy volunteers. Physiological Measurement.

[21] Guzik P (2010). Asymmetric features of short-term blood pressure variability. Hypertension Research.

[22] Costa M, Goldberger AL, Peng C-K (2005). Multiscale entropy analysis of biological signals. Phys. Rev. E.

[23] Peng C-K (1994). Mosaic organization of dna nucleotides. Phys. Rev. E.

[24] Arneodo A, Bacry E, Muzy J (1995). The thermodynamics of fractals revisited with wavelets. Physica A: Statistical Mechanics and its Applications.

[25] Serrano E, Figliola A (2009). Wavelet leaders: A new method to estimate the multifractal singularity spectra. Physica A: Statistical Mechanics and its Applications.

[26] Brignole M (2000). New classification of haemodynamics of vasovagal syncope: beyond the vasis classification. Europace.

[27] Makowiec D, Graff B, Struzik ZR (2017). Multistructure index in revealing complexity of regulatory mechanisms of human cardiovascular system at rest and orthostatic stress in healthy humans. Physica A: Statistical Mechanics and its Applications.

[28] Hainsworth, R. *Physiological background of heart rate variability*, 1–12 (Blackwell Publishing, 2007).

[29] Porta A (2012). Short-term complexity indexes of heart period and systolic arterial pressure variabilities provide complementary information. Journal of Applied Physiology.

[30] Eckberg DL (1983). Human sinus arrhythmia as an index of vagal cardiac outflow. Journal of Applied Physiology.

[31] Davis MJ, Hill MA (1999). Signaling mechanisms underlying the vascular myogenic response. Physiological Reviews.

[32] Ramchandra R, Barrett CJ, Malpas SC (2005). Nitic oxide and sympathetic nerve activity in the control of blood pressure. Clinical and Experimental Pharmacology and Physiology.

